# Predictive biomarkers in gastric cancer

**DOI:** 10.1007/s00432-022-04408-0

**Published:** 2022-10-19

**Authors:** C. Röcken

**Affiliations:** Department of Pathology, Christian-Albrechts-University, Arnold-Heller-Str. 3, Haus 14, Haus U33, 24105 Kiel, Germany

**Keywords:** Gastric cancer, Biomarker, Receptor tyrosine kinase, Immune checkpoint inhibitor, Precision medicine

## Abstract

Predictive biomarkers are the mainstay of precision medicine. This review summarizes the advancements in tissue-based diagnostic biomarkers for gastric cancer, which is considered the leading cause of cancer-related deaths worldwide. A disease seen in the elderly, it is often diagnosed at an advanced stage, thereby limiting therapeutic options. In Western countries, neoadjuvant/perioperative (radio-)chemotherapy is administered, and adjuvant chemotherapy is administered in the East. The morpho-molecular classification of gastric cancer has opened novel avenues identifying Epstein–Barr-Virus (EBV)-positive, microsatellite instable, genomically stable and chromosomal instable gastric cancers. In chromosomal instable tumors, receptor tyrosine kinases (RKTs) (e.g., EGFR, FGFR2, HER2, and MET) are frequently overexpressed. Gastric cancers such as microsatellite instable and EBV-positive types often express immune checkpoint molecules, such as PD-L1 and VISTA. Genomically stable tumors show alterations in claudin 18.2. Next-generation sequencing is increasingly being used to search for druggable targets in advanced palliative settings. However, most tissue-based biomarkers of gastric cancer carry the risk of a sampling error due to intratumoral heterogeneity, and adequate tissue sampling is of paramount importance.

## Introduction

Gastric adenocarcinoma (GC) is the fifth most common cancer and the third most common cause of cancer-related deaths, accounting for almost 800.000 deaths world-wide (Smyth et al. 2020). The incidence and prevalence of GC varies geographically, and its prevalence is twice as high among men than in women. Emerging data show that East Asia, Central Europe, and Eastern Europe have the highest rates of GC, collectively accounting for 87% of all new cases registered worldwide. In Africa and North America, significantly lower rates have been observed (Smyth et al. 2020). During the past 30–50 years, the standardized incidence rates of non-cardia GC have declined worldwide. However, cancer of the proximal stomach/cardia and esophagogastric junction has either been stable or even increased. Typically, the incidence of GC is predominantly observed in the elderly, often peaking at the 7th and 8th decade of life; moreover, with a rapidly aging population, the prevalence of GC is relatively high (Smyth et al. 2020). In regions reporting low incidence of the disease, it is diagnosed at an advanced stage, thereby restricting targeted therapeutic treatment options, with patient prognosis remaining rather dismal. The disease specific 5 year survival rate for both sexes ranges between 30% and 35%.

## Etiology

Gastric adenocarcinoma has a diverse etiology, and the main risk factors include dietary factors, such as high salt intake, tobacco consumption, and Helicobacter pylori (H. pylori) infection (Smyth et al. 2020). Correa proposed a model for gastric carcinogenesis: chronic atrophic gastritis leads to intestinal metaplasia, dysplasia, and finally GC (Correa 1992). In recent years, multiple bacterial virulence factors have been identified for colonization, persistence, ulcers, and cancer risk, such as CagA, DupA, IceA1, NapA, oipA, and VacA (Malfertheiner et al. 2014). Host factors, e.g., polymorphisms in genes coding for pro- and anti-inflammatory cytokines, environmental factors, and socioeconomic status further modulate the individual cancer risk. Proximal GC and esophagogastric junction carcinoma are associated with gastroesophageal reflux disease and Barrett’s mucosa (Smyth et al. 2020).

Approximately 10% of all GC cases have a familial or hereditary trait (Smyth et al. 2020). In familial GC, the main predisposing factors include H. pylori infection, dietary habits, and gene polymorphisms. The underlying germ line mutations causing hereditary type GC are found in 1–3% of all cases (Moehler et al. 2019). Genes which have been linked with hereditary type GC are *CDH1* (coding for E-cadherin), *CTNNA1* (α-E-catenin) (Majewski et al. 2013; Petrovchich et al. 2016), *FBXO24* (F-Box-protein 24), *DOT1L* (DOT1-like histone H3K79 methyltransferase), *INSR* (insulin receptor), *MAP3K6* (mitogen-activated protein kinase 6), *PRSS1* (protease serine 1) (Donner et al. 2015; Petrovchich et al. 2016), and mutations in the APC promoter 1 B (Worthley et al. 2012; Li et al. 2016). GC can also arise in a setting of other well defined hereditary cancer syndromes, such as familial adenomatous polyposis (*APC*), Cowden syndrome (*PTEN*), Lynch syndrome (*hMLH1*, *hMLH2*), juvenile polyposis (*BMPR1A*), MUTYH-associated adenomatous polyposis (*MUTYH*), Li-Fraumeni syndrome (*TP53*), Peutz–Jeghers syndrome (*STK11*), and hereditary breast and ovarian cancer (*BRCA1*/*2*) (van der Post et al. 2015).

## Histology of gastric cancer

**Fig. 1:**
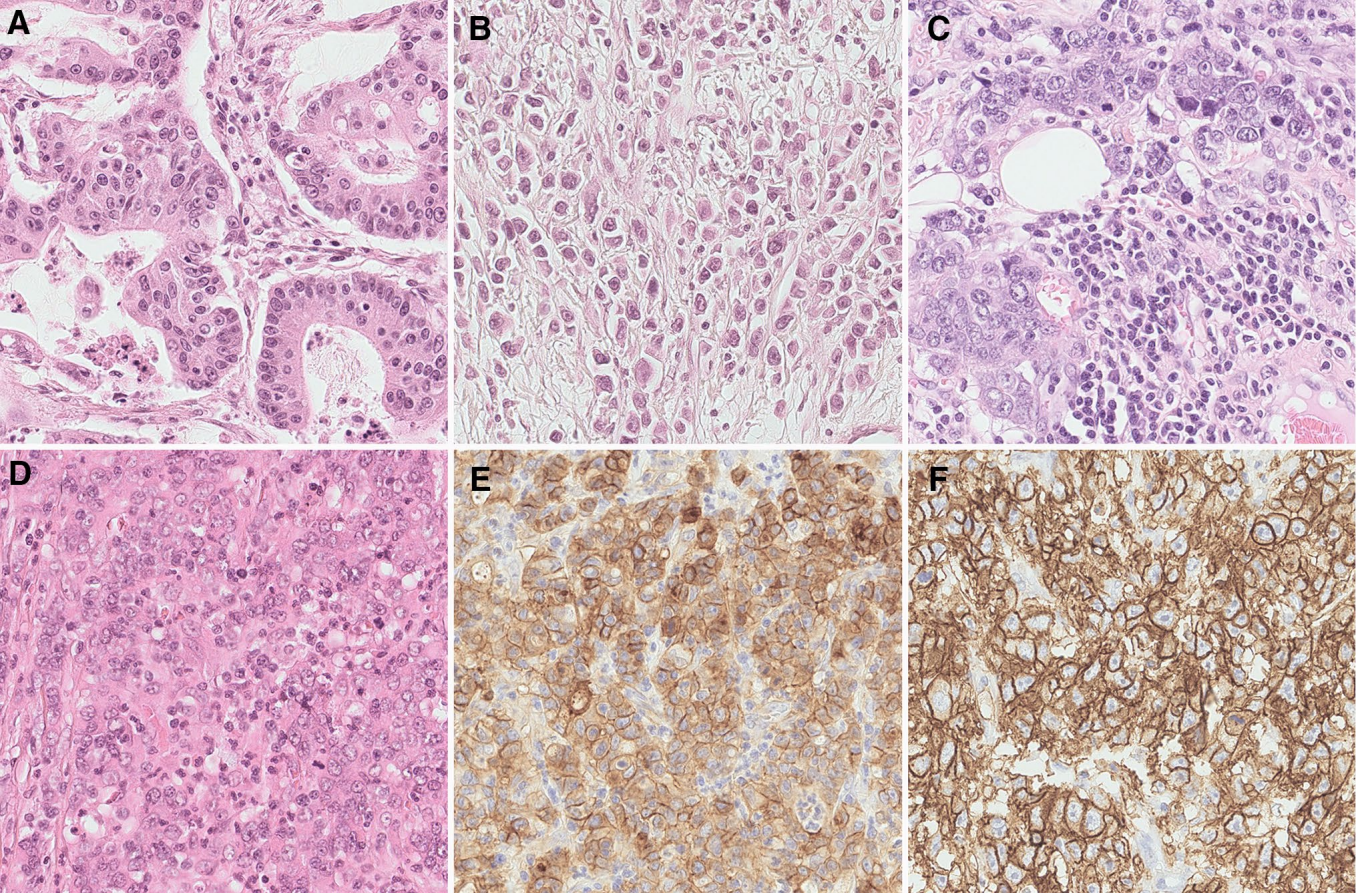
Histology of gastric cancer. Intestinal type (**A**), diffuse type (**B**), Epstein–Barr-virus positive (**C**) and microsatellite instable (**D**) gastric cancer. Note the tumor-associated inflammatory reaction (**C**). The microsatellite instable gastric cancer shows expression of PD-L1 (**E**). An example of immunostaining for Claudin 18.2 is shown in (**F**). Hematoxylin and eosin-staining (**A**–**D**), anti-PD-L1-antibody (**E**), anti-Claudin 18.2-antibody (**F**). Original magnifications: 400-fold

The World Health Organization (WHO) subclassifies adenocarcinomas of the stomach into tubular, parietal cell, mixed type, papillary, micropapillary, mucoepidermoid, mucinous, poorly cohesive (including signet-ring cell carcinoma), medullary carcinoma, hepatoid adenocarcinoma, and Paneth cell carcinoma (Board 2019). In spite of that, the Laurén classification is still used in cancer research and many clinical trials (Fig. 1) (Lauren 1965). Laurén classified GC into intestinal, diffuse, mixed, and unclassified types (Lauren 1965). Most GCs (> 90%) are adenocarcinomas. Molecular characterization studies have been conducted and applied to Laurén’s classification (Wang et al. 2014). The morpho-molecular classification system for GC (see below) corresponds only rudimentarily with the current WHO classification (Cancer Genome Atlas Research 2014; Smyth et al. 2020).

## Molecular subtypes of gastric cancer

The Cancer Genome Atlas Research Network had performed an integrative genomic analysis and proposed four molecular subtypes, which can be used to stratify patients and design clinical trials for targeted therapy: Epstein–Barr virus-associated (EBV), microsatellite instable (MSI), chromosomal instable (CIN), and genomically stable (GS) GC (Cancer Genome Atlas Research 2014; Wang et al. 2014). These studies linked distinct molecular subtypes with some histological phenotypes. It forms the basis for novel therapeutic strategies and precision medicine (Cancer Genome Atlas Research 2014, Wang et al. 2014).

### Microsatellite instability in gastric cancers (MSI-GC)

MSI-GCs are characterized by a very high mutational load and CpG-island methylator phenotype (CIMP). The DNA mismatch repair protein MLH1 is frequently silenced in these tumors. MSI-GCs account for 0–44.5% of all GC cases (Cortes-Ciriano et al. 2017; Mathiak et al. 2017; Guan et al. 2021). They are predominantly observed in elderly patients and located in the distal stomach. MSI-GCs harbor significantly lower numbers of lymph node metastases, and have a better overall and tumor-specific survival (Cortes-Ciriano et al. 2017; Mathiak et al. 2017; Smyth et al. 2017; Guan et al. 2021; Quaas et al. 2021a, b). Different from sporadic microsatellite instable colorectal cancer, MSI-GCs lack *BRAF*-mutations (Cancer Genome Atlas Research 2014) and *BRAF* cannot be used as predictive biomarker like in malignant melanomas (Warneke et al. 2013a, b).

Frequently, MSI-GCs exhibit rare and indicative histological phenotypes (Fig. 1) (Mathiak et al. 2017) and an abundant tumor-associated inflammatory cell infiltration comprising either neutrophils and/or lymphocytes is commonly noted, with little or no desmoplastic stromal components (Mathiak et al. 2017; Smyth et al. 2020).

For two reasons, MSI-GC classification is clinically relevant: (1) MSI-GCs predict improved patient outcomes (Mathiak et al. 2017; Pietrantonio et al. 2019; Guan et al. 2021); (2) MSI-GCs frequently express immune checkpoint molecules such as PD-L1 (Fig. 1) making them eligible for treatment with immune checkpoint inhibitors (Cancer Genome Atlas Research 2014; Böger et al. 2016; Pietrantonio et al. 2021). MSI-GCs can be classified and identified using antibodies directed against MLH1, PMS2, MSH2, and MSH6, and mononucleotide markers BAT-25, BAT-26, NR-21, NR-24, and NR-27 (Mathiak et al. 2017). In most cases, histological phenotypes could raise a doubt (see above). However, as one-third of tumors do not show a particular phenotype, a more general approach may be required for MSI-testing, that is, upfront testing irrespective of the histological phenotype. Most MSI-GCs demonstrate loss of MLH1-expression. Thus, detecting loss of MLH1 by immunostaining may be a cost-effective approach to search for MSI-GC (Gonzalez et al. 2016). However, other members of the DNA-repair machinery, such as MSH2, albeit rarely observed in GC, can be lost and may indicate Lynch syndrome (Matsubayashi et al. 2022).

The significantly better outcome of MSI-GC compared with microsatellite stable GC raised concerns regarding the necessity of neoadjuvant/perioperative (radio-)chemotherapy and adjuvant chemotherapy in this patient subgroup (Mathiak et al. 2017). In support of this notion, an explorative analysis of the MAGIC trial showed that patients with non-metastatic MSI-GC had a better prognosis after surgery compared to those with microsatellite stable GCs. However, MSI-GCs had a worse prognosis when treated with perioperative chemotherapy (median overall survival 9.6 vs. 19.5 months; hazard ratio [HR] 2.18), also showing no major pathological responses to chemotherapy (Smyth et al. 2017; Petrillo et al. 2020). These data were further supported by a large meta-analysis performed by Pietrantonio et al. (Pietrantonio et al. 2019) including individual patient data from four multicenter randomized clinical trials. Again, patients with MSI-GC, who were treated with surgery alone, performed well even without adjunctive chemotherapy. In contrast, patients with MSI-GC, who were treated with chemotherapy (perioperative or adjuvant), did not benefit from this treatment (Pietrantonio et al. 2019). However, these studies suffer from their retrospective nature and large prospective trials are urgently needed.

MSI may also serve as a predictive biomarker for the administration of immune checkpoint inhibitors (Pietrantonio et al. 2021). MSI-GC frequently express PD-L1 (Fig. 1) (Böger et al. 2016; Cho et al. 2021), and anti-PD-1 agents with or without chemotherapy significantly and consistently improved overall survival, progression free survival, and objective response rate vs. chemotherapy alone in the subgroup of patients with advanced MSI-GC (Pietrantonio et al. 2021).

### Epstein–Bar-virus-associated gastric cancer (EBVaGC)

EBVaGC is another molecular subtype, which accounts for 2–20% of all GC cases (Cancer Genome Atlas Research 2014, Wang et al 2014; Böger et al. 2017a, b; Saito et al. 2021), and also shows a CIMP-phenotype. It frequently harbors mutations in *PIK3CA* and *ARID1A*, copy-number amplifications of JAK2 and CD274/PDCD1LG2, and a dysregulation of immune cell signaling molecules. Data show that EBVaGC is more prevalent among Caucasians than Asians, predominantly among males, and younger-aged patients. It occurs primarily in the proximal stomach and postgastrectomy remnant stomach. Multiplicity of EBVaGC is frequently encountered (Fukayama et al. 2011; Saito et al. 2021), and lymph node metastases are less often detected compared with EBV-negative GCs (Tokunaga et al. 1998). In Asian populations, EBVaGC may have a better prognosis than EBV-negative GCs (Liu et al. 2015a, b). EBVaGC and MSI-GC are mutually exclusive (Mathiak et al. 2017). Histologically, EBVaGC may show a tubular and intestinal differentiation. A dense infiltrate of lymphocytes is often noted presenting as an undifferentiated phenotype (lymphoepithelioma-like or medullary) (Fig. 1). Hence, any unusual histological phenotype should be forwarded to EBV-testing. This is best done by EBER-in-situ-hybridization providing a nuclear signal. Immunostaining is less sensitive. GC is commonly of type 1 latency and antibodies directed against EBNA2, LMP1, and ZEBRA may not immunoreact with the tumor cells (Tokunaga et al. 1993; Fukayama et al. 2011). In view of the broad morphological spectrum of EBVaGC, testing should be performed at liberty (Park et al. 2016).

EBVaGCs, like MSI-GC, also express PD-L1 and may respond to immune checkpoint inhibitors (Böger et al. 2016; Wei et al. 2021; Zhang et al. 2021). However, data on the efficacy of immune checkpoint inhibitors in EBVaGC are limited. In a recent review, data from 39 patients were summarized (Wei et al. 2021). Among these 39 patients, 12 had survival information, including progression-free and overall survival. Compared to PD-L1 negative patients, PD-L1 positive patients had superior progression-free survival. Thus, while the identification and diagnosis of EB-VaGC may be clinically relevant, further research is required.

### Chromosomal instable gastric cancers (CIN GC)

Chromosomal instability drives intratumoral heterogeneity, which supports microenvironmental selection and evolution of cancer cell populations, leading to cancer resistance (Tannock et al. 2016). Thus, recognition of CIN GC is highly important. Although, it may not constitute a distinct subgroup but may rather be a compilation of a more heterogeneous group of tumors (Maleki et al. 2017). Currently, there is no validated simple diagnostic method for the identification of CIN besides somatic copy number alteration analysis or defined molecular markers. According to Laurén classification, CIN GC often shows an intestinal phenotype. Genetically, CIN GC are characterized by frequent mutations in the *TP53*-tumor suppressor gene (Cancer Genome Atlas Research 2014). The Cancer Genome Atlas Program identified *TP53* mutations and the loss of its protein’s pathway to be one of its key characteristics; 71% of their CIN tumors had a *TP53* mutation (Cancer Genome Atlas Research 2014). Detection of *TP53* mutations might, therefore, be an accurate method to diagnose CIN in GC. In this context, the immunohistochemical staining of protein p53 was considered a useful diagnostic tool, and some groups tried to classify this type of cancer based on p53 protein status (Cristescu et al. 2015; Gonzalez et al. 2016; Setia et al. 2016). However, our own studies provided evidence that p53-immunostaining is unsuitable to predict *TP53*-mutational status in individual cases and hence CIN GC (Schoop et al. 2020a, b). Other markers might be needed.

Meanwhile, CIN GC also frequently harbors amplifications of genes coding for RTKs, such as *EGFR*, *FGFR2*, *HER2*, and *MET* (Deng et al. 2012; Kiyose et al. 2012), which represent either validated or putative therapeutic targets, and several are being explored in ongoing clinical trials.

#### Human epidermal growth factor receptor 2 (HER2)

**Fig. 2:**
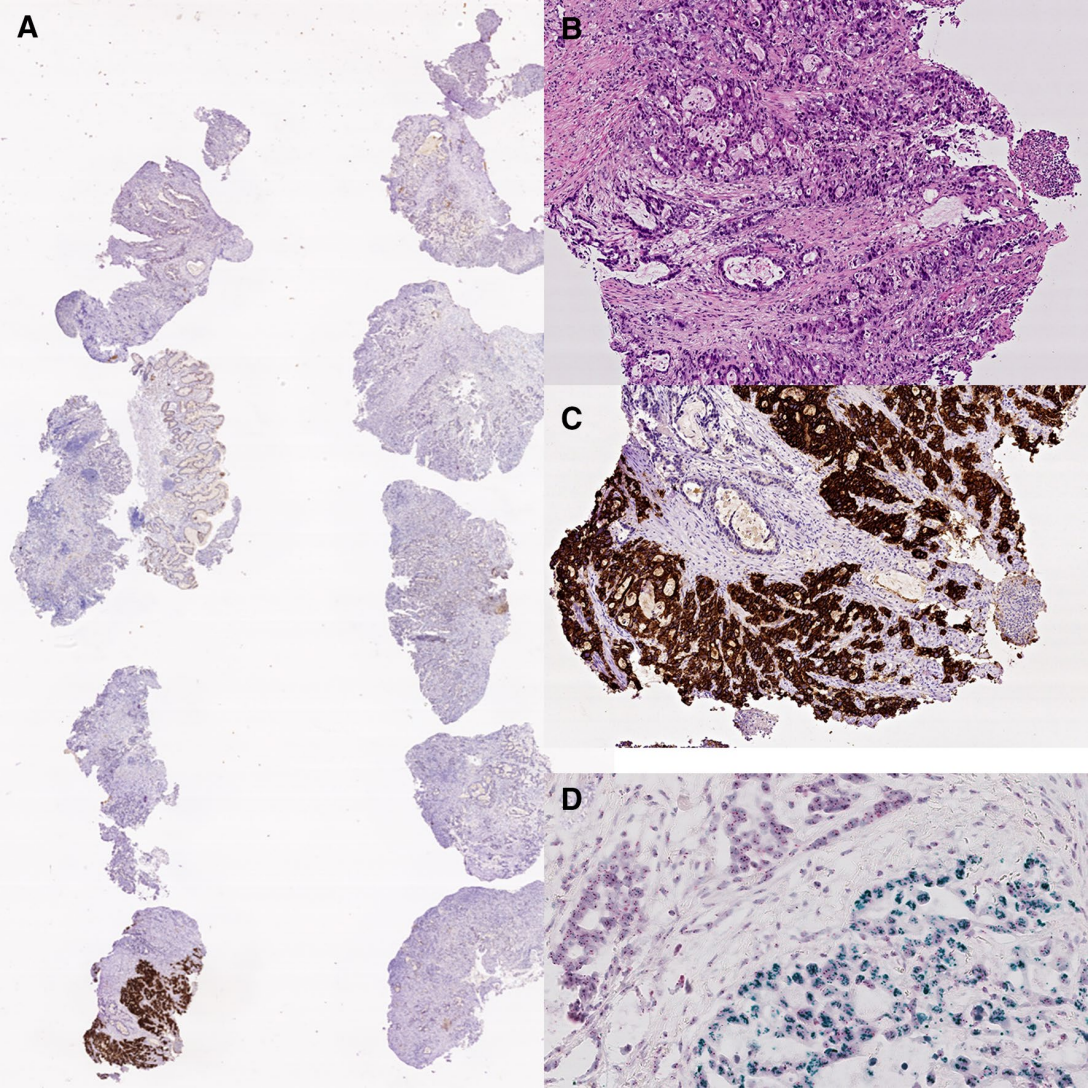
Intratumoral heterogeneity of HER2. Ten biopsy specimens with gastric cancer (**A**) and an intestinal phenotype (**B**) show overexpression of Her2/neu only in a single biopsy specimen (**A**, **C**). Chromogenic in situ hybridization confirms HER2 amplification only in the tumor cells with very strong (3 +) Her2/neu immunostaining (**D**). Hematoxylin and eosin (**B**), anti-Her2/neu-antibody (**A**, **C**), HER2 chromogenic in situ hybridization (**D**). Original magnifications 0.5-fold (**A**), 100-fold (**B**, **C**), 400-fold (**D**)

The European Medicines Agency approved in 2010 trastuzumab, a monoclonal anti-body targeting the human epidermal growth factor receptor 2 (HER2; also known as ERBB2), in combination with chemotherapy for first-line treatment of HER2-positive advanced gastric or esophagogastric junction cancer. The addition of trastuzumab to chemotherapy improved survival in these patients’ cancer compared with chemotherapy alone [13.8 months (95% confidence interval (CI) 12–16) vs. 11.1 months (10–13)] (Bang et al. 2010). Following official approval, testing HER2 became the first predictive biomarker for GC (Ruschoff et al. 2012). Many subsequent studies demonstrated that overexpression of HER2 correlates significantly with HER2-gene amplification. Overexpression of HER2 can be heterogeneously distributed in both, the primary tumor as well as in metastases (Fig. 2), and is more commonly found in proximal and intestinal type GCs, respectively (Warneke et al. 2013a, b; Roviello et al. 2022).

The assessment of HER2-overexpression is complicated and necessitates training as well as expertise (Haffner et al. 2021). The HER2 scoring system of GC is different from breast cancer scoring and also distinguishes biopsy and resection specimens (Ruschoff et al. 2012). HER2 positive GC resection specimens are defined by ≥ 10% of tumor cells showing basolateral immunostaining intensity of 2 + and a confirmatory in situ hybridization showing HER2 amplification or a strong (3 +) basolateral immunostaining. In biopsy specimens, the cutoff is set at ≥ 5 adjacent cancer cells (Ruschoff et al .^2012^). Accurate and validated assay methods should be used and participation in external quality assurance schemes is recommended. The difficulties of accurate assessment of the HER2 status and its impact on patient outcome was demonstrated recently by a prospective multicenter study (VARIANZ) (Haffner et al. 2021). The VARIANZ study enrolled prospectively patients at 35 German sites receiving medical treatment for metastatic GC. Follow-up lasted up to 48 months. HER2 status was assessed centrally using immunohistochemistry and chromogenic in situ hybridization. Central HER2 test results were compared with those obtained previously at local sites: central and local test results were available from 374 patients. In 77 cases (20.6%), central testing revealed HER2-positive GC. However, in 22.7% of patients, the locally assessed HER2 status was not confirmed by central testing. In the majority of these cases, a local HER2 + status was not confirmed centrally, whereas, in nine patients, HER2 was tested positive centrally and negative locally. Most interestingly, patients with centrally confirmed HER2-positive GCs had a significantly longer overall survival when treated with trastuzumab plus chemotherapy as compared with patients who tested negative centrally and positive locally [20.5 months (95% CI 15.7–31.5) vs. 10.9 months (95% CI 8.2–14.4; HR 0.42)] (Haffner et al. 2021). The VARIANZ study also refined the test algorithm, and alternative criteria for HER2-directed treatment were proposed. The mean survival in patients with ≥ 40% of HER2-positive tumor cells was 20.5 months (95% CI 15.6–32.6) vs. 11.4 months (95% CI 9.3–15.0; HR 0.46). The optimized HER2/CEP17 ratio for indicating benefit from trastuzumab was 3.0 with a median survival of 22.8 months (95% CI 16.5–81.7) in patients with a HER2/CEP17 ratio ≥ 3.0 vs. 11.7 months (95% CI 9.3–14.8) for patients with a HER2/CEP17 ratio < 3.0 (HR 0.36) (Haffner et al .^2021^). The VARIANZ study confirms findings made previously by two small Asian studies, in which the effect of homogeneous and heterogeneous expression of HER2 was compared in biopsy and resection specimens (Wakatsuki et al. 2018; Yagi et al. 2019). GCs showing homogeneous overexpression are more likely to respond to HER2-targeted therapy compared with GC overexpressing HER2 heterogeneously (Wakatsuki et al. 2018; Yagi et al. 2019).

Per the HER2 observations and its marked intratumoral heterogeneity (Fig. 2) (Warneke et al. 2013a, b), experts from an interdisciplinary German group accepted GC-specific HER2 testing protocols and recommended a minimum of five tumor-bearing biopsies from the primary site of the tumor growth (Baretton et al. 2016). Similar recommendations for HER2 testing were released by a group of North American organizations (Bartley et al. 2016). The updated recommendation is testing multiple biopsy fragments from either a primary tumor metastasis site or from the resected primary tumor. In the case of biopsy specimens, the present protocol recommends a minimum of five biopsy specimens, optimally, six to eight are required for interpreting intratumoral heterogeneity and providing necessary tumor material for diagnosis and biomarker testing (Bartley et al. 2016).

#### Hepatocyte growth factor receptor (MET)

MET has pleiotropic effects and induces proliferation, survival, motility, cell scattering, angiogenesis, tubulogenesis, drives epithelial–mesenchymal transition and tumor invasion (Graveel et al. 2013; Hack et al. 2014). Most commonly, the MET pathway is activated in GC by protein overexpression, which can be detected by immunohistochemistry and occurs in 50–65% of cases. MET can be overexpressed in pre-cancerous intestinal metaplasia and dysplastic lesions, underscoring its critical role in gastric carcinogenesis. It is frequently found in well-differentiated tubular adenocarcinoma (67%), intestinal-type (35%), and less commonly in diffuse-type GCs (15–51%). Overexpression of MET has been linked to a more aggressiveness phenotype, i.e., advanced local tumor growth, nodal spread, distant metastasis, advanced tumor stage, recurrence, and poor survival (Metzger et al. 2016). MET can also be activated by gene amplification and, although infrequent, co-amplification with other RTKs can occur in GC. Other activating genetic mutations of MET remain exceedingly rare in GC (for a review see (El Darsa et al. 2020)).

MET was also explored as druggable target in GC (Lennerz et al. 2011; Shah et al. 2013; Teng et al. 2013; Hack et al. 2014; Jardim et al. 2014; Kang et al. 2014), and efficacy may depend on its expression pattern detected by immunohistochemistry and/or in situ hybridization (Hack et al. 2014). Many studies investigated the tumor-biological and clinicopathological characteristics of MET-positive GCs. The prevalence ranged from 3.8 to 85% (Drebber et al 2008; Betts et al. 2014). In our study, any immunostaining of MET, i.e., weak, moderate or strong, was observed in 192 (42.1%) cases (Metzger et al. 2016). The wide spectrum of immunopositivity in the various studies stems from the usage of different types of antibodies and different, non-standardized scoring systems. In fact, until now, no standardized scoring system was established for MET in GC. Furthermore, like HER2, MET shows intratumoral heterogeneity: amplified and unamplified tumor cell clones occur in the same tumor distinguishable on a cell-by-cell level (Catenacci et al. 2011; Nagatsuma et al. 2015; Metzger et al. 2016), further compromising its usage as a predictive biomarker. However, diverse MET inhibitors have been developed and are also currently under investigation in clinical trials (El Darsa et al. 2020). Until now, none has reached formal approval.

#### Fibroblast growth factor receptor (FGFR2)

The dysregulation of the fibroblast growth factor receptor (FGFR) pathway has been studied as therapeutic target in many different tumor types (Babina et al. 2017). Activation of FGFR signaling is caused by gene amplification, activating mutation, and chromosomal translocations/fusions. In GC, *FGFR2* is most frequently mutated among the FGFR family members occurring in approximately 4.0% of the cases with advanced GC. The frequencies of amplifications, mutations, translocations/fusions, and multiple alterations among FGFR2-dysregulated GCs are given as 72, 13, 8.6, and 6.3%, respectively (Ooki et al. 2021) and gene amplification is the common mechanism of FGFR2 overexpression. So far, no association has been established between *FGFR2*-amplification and gender, anatomical site, histological subtype, or TNM classification (Deng et al. 2012; Silva et al 2018). *FGFR2*-amplification is not limited to CIN GCs, it was detected in genomically stable GCs (Schrumpf et al. 2022).

The protein can be detected in the cytoplasm and at the cell membrane in both, intestinal and diffuse type GC (Schrumpf et al. 2022). There are two major FGFR2 isoforms, i.e., FGFR2-IIIb and IIIc, which are determined by alternative splicing of a ternary extracellular immunoglobulin (Ig) domain III. In GC, FGFR2-IIIb is the predominantly overexpressed isoform (Ooki et al. 2021). Ueki et al. were the first to report on the potential prognostic significance of FGFR2 in GC (Ueki et al. 1995). *FGFR2* gene amplification occurs in 2–9% of the cases (Jung et al. 2012; Matsumoto et al. 2012; Betts et al. 2014; Cancer Genome Atlas Research 2014; Su et al. 2014; Seo et al. 2017; Hur et al. 2020) and has been shown to be an independent prognostic factor for patient survival (Su et al 2014). Overexpression was found in up to 60% of the patients (Tokunaga et al. 2016; Hosoda et al. 2018), the significance of which has been explored in a number of studies. Matsunoba et al. linked high expression with favourable outcome (Matsunobu et al. 2006). To the contrary, four Asian studies linked FGFR2 overexpression to poor overall and tumor-specific survival (Murase et al. 2014; Nagatsuma et al. 2015; Ahn et al. 2016; Hosoda et al. 2018). Inokuchi et al. demonstrated prognostic significance of FGRR2 overexpression only in diffuse-type GC (Inokuchi et al 2017). Recently, a meta-analysis provided evidence that FGFR2 overexpression is associated with greater depth of tumor invasion, higher rates of lymph node metastasis, more advanced disease stage and worse outcome (Kim et al. 2019). However, FGFR2 protein expression in GC was most commonly studied in Asian populations, and data on White patients are scarce (Kim et al. 2019; Schrumpf et al. 2022).

In our own study on FGFR2 in a Western cohort, protein expression detected by immunohistochemistry did not correlate with patient outcome. However, using different cutoff values, a negative correlation between FGFR2-expression and patient survival was found for diffuse type GC. FGFR2 expression was associated with lower tumor grade and intestinal phenotype (*p* ≤ 0.0001). FGFR2-positive diffuse type GCs classified as a small subset of patients with a poor tumor specific survival (5.29 ± 1.3 vs. 14.67 ± 1.9 months; *p* = 0.004) (Schrumpf et al. 2022).

FGFR2 is currently explored for the treatment of GC; however, no standardized test algorithm has been developed yet, and no drug has passed formal approval by the European Medicines Agency for GC treatment.

#### Epidermal growth factor receptor (EGFR)

The epidermal growth factor receptor (EGFR) is frequently mutated in diverse types of carcinomas, including GC. The signaling pathway consists of several overlapping and interconnecting networks including the phosphatidylinositol 3-kinase (PI3K)/Akt (PKB) pathway, the Ras/Raf/MEK/ERK1/2 pathway, and the phospholipase C (PLCγ) pathway. Overexpression and/or gene amplification of EGFR/*EGFR* are found in 2–35% of GCs and significantly impact patient prognosis and survival rate. The EGFR-amplified GCs show a preponderance of male patients and affect the distal stomach (Park et al. 2016). EGFR has been and is still being explored for the treatment of GC. Again, no standardized test algorithm has been developed, and no drug has reached routine clinical application.

Ramucirumab is a human IgG1 monoclonal antibody that targets VEGF receptor 2. However, no companion diagnostics is required prior to its administration (Fuchs et al. 2014; Nakamura et al. 2021).

### Genomically stable gastric cancers

Genomically stable GCs are characterized by a diffuse histological phenotype according to Laurén classification. They frequently harbor mutations in *CDH1* and *RHOA*, and show rearrangements between *CLDN18* and *ARHGAP26* or *ARHGAP6* (Cancer Genome Atlas Research 2014; Kakiuchi et al. 2014). However, apart from CLDN18 mutations, Claudin 18.2 is also currently explored as therapeutic target in GC irrespective of the molecular subtype.

#### Claudin 18.2 (CLDN18.2)

CLDN18.2, a member of the claudin family, is a component of tight junctions, regulating paracellular barrier functions (Oshima et al. 2013). The expression of the isoform 2 of CLDN18.2 (CLDN18.2) is restricted to differentiated epithelial cells of the gastric mucosa and primary GC (Fig. 1), underscoring its potential as druggable target. Ectopic expression is also commonly detected in other tumor types, such as lung, esophageal, pancreatic, and ovarian cancer (Sahin et al. 2008). A limited number of studies explored CLDN18.2 in GC. In a Japanese study, moderate-to-strong CLDN18.2 expression [≥ 2 + membrane staining intensity in ≥ 40% of tumor cells (FAST eligibility criterion; see below)] was observed in 52% of primary tumors and 45% of lymph node metastases. Expression was significantly higher in GCs of the diffuse type according to Lauren and in high grade (G3) tumors (Rohde et al. 2019). Moentenich et al. detected CLDN18.2 in 18.4% of their cases (Moentenich et al. 2020). No correlations were found between expression and clinicopathological data (sex, age, local tumor growth, nodal spread, and tumor grade). However, a significantly decreased expression was observed in tumor types with upregulated HER2 expression. Neoadjuvant treatment had no impact on the expression (Moentenich et al. 2020). Arnold et al. observed a high expression of CLDN18.2 in 17.1% of their primary tumors, in 26.7% of lymph nodes, and 16.7% of distant metastasis (Arnold et al. 2020). Expression in lymph node metastasis and primary tumors correlated significantly. High expression did not correlate with histological phenotype, tumor stage, or overall survival (Arnold et al. 2020). In our study, the expression of CLDN18.2 correlated with mucin phenotype, EBV-status, the integrin αvβ5, the EpCAM extracellular domain EpEX and lysozyme. CLDN18.2 status did not correlate with Laurén phenotype, survival, or any other clinicopathological patient characteristic (Dottermusch et al. 2019). These conflicting results largely stem from different antibodies, staining, and scoring systems. However, harmonization is to be expected in the near future due to ongoing clinical trials.

The phase II-FAST study investigated CLDN18.2 tumor expression and therapy with the chimeric monoclonal anti-CLDN18.2 antibody zolbetuximab in combination with first line chemotherapy (EOX: epirubicin + oxaliplatin + capecitabine) in patients with advanced cancer of the esophagogastric junction and stomach and a moderate to strong expression of CLDN18.2 in ≥ 40% tumor cells. Both progression free survival (95% CI 0.29–0.67; HR 0.44;) and overall survival (95% CI 0.39–0.77; HR 0.55) were significantly improved with zolbetuximab + EOX compared with EOX alone. This significant progression free survival benefit was retained in patients with moderate to strong CLDN18.2 expression in > 70% of tumor cells (95% CI 0.23–0.62; HR 0.38) (Sahin et al. 2021).

## Next generation sequencing

Molecular tumor boards are now increasingly used to search for druggable targets by next generation sequencing (NGS), which may also include GC (Hoefflin et al. 2021). These studies are often done on small biopsy specimens or a limited number of tissue samples. However, a few studies illustrate the substantial intraprimary and intermetastatic genetic heterogeneity of GC (Röcken et al 2021). A substantial variation in the extent of mutational overlap or mutational heterogeneity between primary and lymph node metastasis genomes was found by Lee et al. in 15 pairs of primary GC and their matched lymph node metastases, which were studied by whole-exome sequencing (Lee et al. 2019). Pectasides et al. studied two independent patient cohorts (Pectasides et al 2018). In the first cohort, a single biopsy sample was obtained from the primary tumor of 11 patients and was compared with biopsies from synchronous metastates. In a second cohort, more than 100 samples obtained from the primary tumors and metastatic sites of 26 patients were forwarded to targeted sequencing (Pectasides et al. 2018). Discrepant pathogenic alterations between primary tumors and paired metastatic lesions were found in 45% of the patients. With regard to RTKs, 9 of 12 cases (75%) were discordant across all matched samples (Pectasides et al. 2018). Four MSI GCs were forwarded to multiregional sequencing by Loga et al. An extreme intratumoral heterogeneity as well as evidence of parallel evolution in this special subtype was discovered (von Loga et al. 2020).

We performed multiregional sequencing in nine GCs and harbored 16,537 non-synonymous mutations (Röcken et al. 2021). Intratumoral heterogeneity of somatic mutations and copy number variants were present in all tumors. 53–91% of the non-synonymous mutations were not present in each patient’s sample; 399 genes harbored 2–4 different non-synonymous mutations in the same patient; 175 genes showed copy number variations, the majority being heterogeneous, including CD274 (PD-L1). Multisample tree-based analyses provided evidence for branched evolution being most complex in a MSI GC (Röcken et al. 2021). Collectively, these data illustrate the risk of misinterpreting tumor genetics in GC based on single sample analysis. Thus, when NGS is utilized, caution must be taken regarding the validity and significance of the findings.

## Immune checkpoint molecules

In their seminal updated review on the Hallmarks of Cancer, Hanahan and Weinberg added immune evasion as a strategy of malignant tumors to escape destruction by the immune system (Hanahan et al. 2011). Observational, experimental, and clinical data strongly support the importance of the immune system in combating tumor development and progression (Hanahan et al. 2011). As a result, checkpoint inhibitors gained considerable attention and are now widely explored and used as novel treatment options in cancer, including GC (Bolandi et al. 2021).

The B7 family of immune checkpoint molecules encompasses eleven members: B7-1, B7-2, B7–H1 (PD-L1), B7-DC (PD-L2), B7–H2, B7–H3, B7–H4, B7–H5 (VISTA), B7–H6, B7–H7, and Ig-like domain-containing receptor 2 (ILDR2). The interaction of the B7 family of immune-regulatory ligands with their corresponding receptors induces and inhibits T cell responses by sending co-stimulatory and co-inhibitory signals, respectively (Bolandi et al. 2021). Several of these members are explored as druggable targets or have already been successfully implemented as such in patient care. However, efficacy of immune checkpoint inhibitors varies between patients and patient selection is of crucial importance here as well.

### PD-L1

PD-L1 (B7-H1) is a 290 amino acid type I transmembrane surface glycoprotein. It is encoded by CD274, which is located on chromosome 9. Several cell types of the immune system express PD-L1, such as lymphocytes and dendritic cells. Aberrant expression is observed in diverse solid tumors. PD-L1 is the ligand of programmed cell death 1 (PD-1), another member of the immunoglobulin superfamily B7. PD-1 is expressed by activated T-cells on the germinal center of lymph follicles, tumor infiltrating lymphocytes and other immune cells (Keir et al. 2008) and involved in immunemodulation (Freeman et al. 2000). Binding of PD-L1 to PD-1 suppresses T-cell receptor signaling. This in turn down regulates the immune response and enables cancer cells to escape the destruction by the immune system (Zou et al. 2008). Administration of PD-1/PD-L1 checkpoint inhibitors target the PD-1/PD-L1 interaction and restore cancer cell-directed immune response (Poole 2014). PD-L1 is expressed by GCs and is significantly more prevalent in men, GCs of the proximal stomach, un-classified, papillary, Her2/neu-positive, EBVaGc, and MSI-GC (Böger et al. 2016).

In diagnostic surgical pathology, immunohistochemistry is used to assess the PD-L1 status by applying the tumor proportion score (TPS; percentage of PD-L1 positive tumors cells) and the combined positivity score [CPS; number of PD-L1 staining cells (tumor cells, lymphocytes, macrophages) divided by the total number of viable tumor cells, multiplied by 100]. Although CPS can exceed 100, the maximum score is defined as CPS 100.

Recently, the CheckMate-649 study showed that nivolumab, an anti-PD-1-antibody, in combination with chemotherapy significantly improved overall survival (98.4% CI 0.59–0.86; HR 0.71) and progression-free survival (98% CI 0.56–0.81; HR 0.68) vs. chemotherapy alone in patients with a PD-L1 CPS ≥ 5 and advanced gastric, esophagogastric junction, and esophageal adenocarcinoma (Janjigian et al. 2021). Nivolumab was also shown to be efficacious in resected esophageal or esophagogastric junction cancer after neoadjuvant chemoradiotherapy (Kelly et al. 2021). The KEYNOTE-590 study explored the efficacy of prembolizumab, another anti-PD-1 antibody, in advanced esophageal and Siewert type 1 esophagogastric junction adenocarcinomas. Overall survival was longer in the pembrolizumab plus chemotherapy group than in the placebo plus chemotherapy group [median 11.6 months (95% CI 9.7–15.2) vs. 9.9 months (95% CI 0.54–1.02); HR 0.74] (Sun et al. 2021).

Based on these data, pembrolizumab in combination with platinum and fluoropyrimidine based chemotherapy, was approved for the first-line treatment of patients with locally advanced unresectable or metastatic carcinoma of the esophagus or HER2 negative esophagogastric junction adenocarcinoma in adults whose tumors express PD-L1 with a CPS ≥ 10 (https://www.ema.europa.eu/en/medicines/human/EPAR/keytruda). Nivolumab in combination with fluoropyrimidine- and platinum-based combination chemotherapy was granted approval by the European Medicines Agency for first-line treatment of adult patients with HER2-negative advanced or metastatic gastric, esophagogastric junction cancer or esophageal adenocarcinoma whose tumors express PD-L1 with CPS ≥ 5 (https://www.ema.europa.eu/en/medicines/human/EPAR/opdivo).

Following HER2, PD-L1 is the second predictive biomarker for GC. It has to be tested before treatment with approved immune checkpoint inhibitors can be administered. In general, PD-L1 scoring is sensitive to antibody selection, staining protocols, and expertise in the assessment of immunostaining (Munari et al. 2018; Ahn et al. 2021; Narita et al. 2021; Noske et al. 2021a, b; Noske et al. 2021a, b). However, the interchangeability of PD-L1 assays in GC has been demonstrated (Ahn et al. 2021; Narita et al. 2021).

### VISTA

V-domain immunoglobulin (Ig)-containing suppressor of T-cell activation (VISTA) is a 311 amino acid type I-membrane protein. Various hematopoietic cells, such as myeloid, granulocytic, and T cells, express predominantly VISTA (Wang et al. 2019). P-selectin glycoprotein ligand 1 (PSGL-1) and V-Set and Immunoglobulin domain containing 3 (VSIG3) were proposed as binding partner(s). In addition, VISTA may function both as a ligand (for antigen presenting cells) and a receptor (for T cells). It suppresses T cell activation. In murine tumor models, monoclonal antibodies targeting VISTA boost antitumor immunity by increasing the number and elevating the function of intratumoral T cells (Le Mercier et al. 2014). It is noteworthy that VISTA-induced T cell activation appears to be nonredundant from the PD-1/PD-L1 pathway. Thus, a blockade of both, VISTA and PD-1, might open novel avenues for cancer treatment, as it was shown in murine tumor models (Liu et al. 2015a, b; Kondo et al. 2016). Recently, one phase I study using an anti-VISTA monoclonal antibody (JNJ-61610588; NCT02671955) and a phase I study that targets both VISTA and PD-L1/PD-L2 in solid tumors using a small molecule (CA-170; NCT02812875) have started.

Data on VISTA are limited. Böger et al. (Böger et al. 2017a, b) showed that the VISTA expression was associated with the tumor localization, Laurén phenotype, EBV, *KRAS*- and *PIK3CA*-mutational status, and PD-L1 expression. However, no significant correlation was observed with patient outcomes. A change in immune cell expression of VISTA during tumor progression was observed (Böger et al. 2017a, b). Loeser et al. (Loeser et al. 2019) observed strong positive outcomes for VISTA-positive tumors in the pT1/T2 stages, with lower expression levels of VISTA in pT3/T4 tumor samples. However, the expression of both PD-L1 and VISTA is sensitive to neoadjuvant (radio-) chemotherapy and is associated with poor tumor regression. Schoop et al. compared a cohort of therapy naïve GCs with a cohort of neoadjuvantly/perioperatively treated GCs. They found a major increase in overall immune cell density coupled with an over proportional increase in PD-1 and VISTA positive immune cells in neoadjuvantly/perioperatively treated GCs (Schoop et al. 2020a, b). The frequency of VISTA expression in tumor cells also substantially increased. In contrast, PD-L1 expression was decreased in immune cells and tumor cells of neoadjuvantly treated GCs (Schoop et al. 2020a, b). Currently, two phase 1/2 clinical trials are listed at www.clinicaltrials.gov, exploring monoclonal antibodies targeting VISTA in solid tumors.

### B7–H3

B7 homolog 3 protein (B7–H3, or CD276), a 534 amino acid protein, is another member of the B7 family of immune checkpoint molecules involved in immune evasion. The exact receptor of B7–H3 is currently unknown (Castellanos et al. 2017). It is overexpressed in various cancers (Castellanos et al. 2017; Ni et al. 2017) and may inhibit CD8 + T cells (Lee et al. 2017). B7–H3 is found both in tumor cells and the tumor immune microenvironment, i.e., endothelial cells, fibroblasts, B-lymphocytes, macrophages, natural killer cells, and dendritic cells (Zhan et al. 2019). B7–H3 seems to be linked to cancer progression, metastatic behavior, and worse prognosis in several cancers including cancers of the lung, breast, prostate, kidney, and colon (Ni et al. 2017). So far, only a few studies examined the expression of B7–H3 in relation to the distribution of CD8 + T cells in GC (Guo et al . 2019). Again, data are scarce. Recently, we used double immunohistochemical staining to study the spatial distribution of CD8 + T cells in relation to B7–H3 positive cells. B7–H3 was expressed mainly in the tumor stroma of GC (76% of all cases). GCs with high expression of B7–H3 showed larger spatial differences of CD8 + T cells (86.4/mm2 in tumor center vs. 414.9/mm2 in invasive front) compared to the B7–H3-low group (157.7/mm2 vs. 218.7/mm2, respectively) (Ulase et al. 2021).

Several trials are listed in www.clinicaltrials.gov targeting B7–H3. None has reached clinical application, yet.

### Sexual dimorphism

The susceptibility of GC shows a striking sex-specific difference. According to the European Network of Cancer Registries, the estimated GC incidence in men is almost double that of women. This also applies to mortality, with an estimated 63,600 gastric cancer deaths in men and 43,700 in women (ENCR Factsheet Stomach Cancer; https://ec.europa.eu/jrc/en/science-update/new-factsheet-stomach-cancer-europe-released). These differences are unlikely related to H. pylori infection, the major risk factor for GC (Group 2001; Brusselaers et al. 2017). More importantly, the immune response exhibits sex-specific differences with regard to infectious diseases, vaccination, and autoimmunity. Both, estrogen and androgen exposure influence the effector functions of immune cells (Markle et al. 2014). This sexual dimorphism in immune response capacity is now well recognized. Immune surveillance competence differ between men and women and may contribute to the sex effect observed in malignant tumors (Dorak et al. 2012).

Sex influences the development and progression of cancer (Mauvais-Jarvis et al. 2020), since men and women differ in their immune response (Mirandola et al. 2015; Klein et al. 2016; De Martinis et al. 2020). Therapy response after an immune checkpoint inhibitor therapy is less effective for women (Conforti et al. 2018). One reason is the higher antigenicity in male cancers. To the contrary, the combination of immune checkpoint inhibitors with chemotherapy is less effective for men (Irelli et al. 2020). Response rates most likely depend on the different innate and adaptive immune systems of men and women (Cook et al. 2009; Klein et al. 2016). Despite this evidence, sexual dimorphism in biomedical science is often not specifically addressed and many studies fail to analyze results by sex (Beery et al. 2011). All this possibly also applies to GC. We and others have shown a gender-specific effect for GC (Caruso et al. 2002; Clausen et al. 2020; Quaas et al. 2021a, b). For women with GC, the density of tumor associated neutrophils especially located in the invasion front is an independent predictor of tumor-specific survival. In contrast to men, where no association was found (Caruso et al. 2002; Clausen et al. 2020). Thus, future studies on the application of immune checkpoint inhibitors also need to consider gender as a “tale-telling” biomarker.

## Limitations

This review addresses mainly recent advancements in established and putative predictive biomarkers for tailoring GC treatment. It does not cover research on, e.g., the growing field of long non-coding RNA. So far, research on long non-coding RNA has focused on tumor biology and patient prognosis, rather than predictive biomarker for drug administration.

## Conclusions

Predictive biomarkers are the mainstay of precision oncology. In recent years, major achievements have been made in GC treatment. While targeting HER2 remains the main therapy for a limited number of patients with advanced GC, novel targets have been developed, specifically those addressing immune checkpoint molecules. However, immune oncology must consider sexual dimorphism in tissue-based diagnostics, drug regimens, and patient outcomes. Currently, CLDN18.2 is being explored among several other targets, and further advancements are expected in the near future. The major obstacle to precision medicine for GC is intratumoral heterogeneity, which affects tissue-based diagnostics due to the risk of sampling errors and patient outcomes, and this may likely cause primary and secondary drug resistance.
